# Box C/D snoRNA *SNORD89* influences the occurrence and development of endometrial cancer through 2’-*O*-methylation modification of *Bim*

**DOI:** 10.1038/s41420-022-01102-5

**Published:** 2022-07-05

**Authors:** Hai-juan Bao, Xi Chen, Xin Liu, Wu Wu, Qian-hui Li, Jing-yuan Xian, Yang Zhao, Shuo Chen

**Affiliations:** Department of Obstetrics and Gynecology, Department of Gynecologic Oncology Research Office, Guangdong Provincial Key Laboratory of Major Obstetric Diseases, The Third Affiliated Hospital of Guangzhou Medical University, Guangzhou, 510150 China

**Keywords:** Endometrial cancer, Tumour biomarkers

## Abstract

The small nucleolar RNA (snoRNA) is a type of small non-coding RNA widely distributed in the nucleoli of eukaryotic cells, promoting cancer development. The aim of this study was to assess box C/D snoRNA 89 (*SNORD89*) dysregulations in endometrial cancer. According to the TCGA database as well as the International Federation of Gynecology and Obstetrics (FIGO), higher *SNORD89* expression is found in endometrial cancer tissues. In addition, the *SNORD89* expression level was higher in endometrial carcinoma with lymph node metastasis than in endometrial carcinoma without lymph node metastasis. By interacting with the conservative chaperone protein methylase fibrillarin (Fbl), *SNORD89* inhibits the translation process of the Bim gene, leading to a decrease in Bim protein. Cancer-promoting effect of *SNORD89* can be reversed by Fbl knockdown or Bim overexpressing. What’s more, ASO-mediated silencing of *SNORD89* could inhibit endometrial cancer cell proliferation and migration ability. Taken together, *SNORD89* can modify Bim through 2′-*O*-methylation and affect downstream signaling pathways to promote endometrial cancer occurrence and development. The role of methylation modification in the prevention and treatment of endometrial cancer provides a new understanding and *SNORD89* may be a new diagnostic and therapeutic target for endometrial cancer.

## Abstract (web summary)

Mechanism of action of *SNORD89*

## Graphical abstract

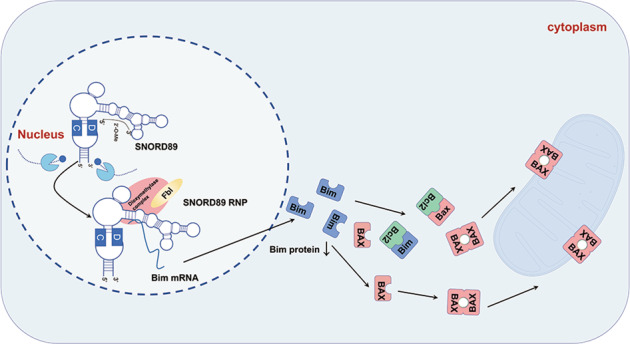

## Introduction

Endometrial cancer is the most common gynecological malignant tumor in developed countries. Its morbidity and mortality also continue to soar [1]. The 5-year relative survival rate of early endometrial cancer is high (96%). However, late diagnosis reduces the 5-year relative survival rate dramatically (18%) [2]. Endometrial cancers with high malignancy and poor prognosis are characterized by the loss of tumor suppressor genes or amplification of multiple oncogenes. Therefore, an in-depth investigation of the molecular mechanism of the occurrence and development of endometrial cancer is essential for the early detection, diagnosis, and treatment of endometrial cancer. The small nucleolar RNA (snoRNA) is a non-coding RNA (ncRNA) [3, 4]. At present, ncRNA is generally believed to potentially regulate various cellular processes, including chromosome remodeling, transcription, translation, and signal transduction [5].

The abnormal expression and dysfunction of ncRNA are also involved in the occurrence and development of multiple malignant tumors, such as liver cancer [6], endometrial carcinoma [7], and colorectal cancer [8]. snoRNA is a type of small ncRNA widely distributed in the nucleus of eukaryotic cells, with a length of 60–300 nt. It can bind to four nucleolar ribonucleoproteins (Fbl/Nop1p, Nop56, Nop58/Nop5p, and P15.5 KD/Snu13p) to form a snoRNP complex and participate in ribosomal RNA (rRNA) processing, RNA shearing, and translation to process regulation and oxidative stress response [9]. Several recent studies have reported that snoRNAs are closely related to cancer occurrence and development. For example, *snoRNA42* overexpression is related to the invasive biological behavior of non-small cell lung cancer (NSCLC), and its expression level is inversely proportional to the overall survival rate of patients, suggesting that *snoRNA42* may become a new prognostic marker and potential therapeutic target of NSCLC [10]. In addition, *snoRNA73A*, *snoRNA73B*, and *snoRNA74A* can activate poly [ADP-ribose] polymerase (*PARP-1*) to regulate downstream signaling pathways and participate in breast cancer development by combining with the DNA repair enzyme *PARP-1* in the absence of DNA damage stimulation [11]. Moreover, *SNORD126* activates the P*I3K*–*AKT* pathway in liver and colorectal cancer by combining with hnRNPK to promote tumor cell proliferation in vitro and tumor formation in xenograft models in vivo [12]. Ravo et al. conducted high-throughput genetic analysis on paired normal, hyperplastic, and cancerous endometrial tissues obtained by endometrial biopsy. They found increased *SNORD116* and *SNORD2* expression, while *SNORD3* was decreased in cancerous endometrial tissues [13]. This suggests that snoRNA is closely related to the occurrence and development of endometrial cancer. However, the specific mechanism of action and possible signaling pathways of snoRNA in endometrial cancer has not yet been reported.

*SNORD89* belongs to the Box C/D snoRNAs in snoRNA, located at 2q11.2, also known as HBII-289. It is characterized by highly conserved boxes, called C (typical motif RUGAUGA, where R is purine) and D (typical motif CUGA). Box C/D snoRNAs could combine with Fbl/Nop1p, Nop56, Nop58/Nop5p, and P15.5 KD/Snu13p to form a snoRNP complex and 2′-*O*-methylate modification of rRNA, transfer RNA (tRNA), and other RNAs, among which Fbl is the key 2′-*O*-methylase of Box C/D snoRNA [14]. Besides, studies have shown that some snoRNAs can be processed into miRNA precursors, forming snoRNA-like microRNAs (sno-miRNAs) similar to snoRNA that plays a miRNA-like role [15]. miRNA-like roles are the same functions played by miRNAs and can silence the expression of target genes. AGO2 is the core element of the RNA-induced silencing complex. It not only promotes the degradation of the target mRNA and inhibits its protein translation in the miRNA/siRNA pathway, but also regulates the biosynthesis and maturation of miRNA, playing a key role in the regulatory pathway [16–18].

According to the function, members of the *Bcl-2* family can be divided into three groups: two groups of antiapoptotic members and one group of proapoptotic members. *Bcl-2* members mostly contain *Bcl-2* tumor domains (BH domains), ranging from BH1 to BH4. *Bim* belongs to the only BH3 proapoptotic group of the *Bcl-2* family, promoting many tumors and cell disorders, such as lung cancer [19], breast cancer [20, 21], osteosarcoma [22], prostatic cancer [23], and melanoma [24]. Various chemotherapeutic drugs contain *Bim* as an intermediary executor of cell death [25, 26]. *Bim* is located in the cytoplasm and can induce apoptosis by directly activating the proapoptotic *Bax*/Bak or by neutralizing the antiapoptotic *Bcl-2* protein (*Bcl-2* and *Bcl-XL*) when activated by foreign signals. When activated directly or indirectly by *Bim*, *Bax* will be inserted into the mitochondrial outer membrane (MOM) and oligomerized with *Bax*/Bak into the pore, enhancing the permeability of MOM and promoting the release of mitochondrial interstitial membrane proteins such as Cytc into the cytoplasm. After Cytc enters the cytoplasm, it can bind to apoptotic protease activator 1 (Apaf-1) to activate Apaf-1 and its oligomer into a sevenfold caspase activation complex, known as Apaf-1 apoptotic body. Apaf-1 can successively recruit and activate caspase-9 and caspase-3 to participate in the hydrolysis of various proteins and finally induce cell apoptosis [27]. In addition to directly activating *Bax* and promoting apoptosis, *Bim* can exert an antiapoptotic effect by antagonizing the antiapoptotic protein *Bcl-2*, which can not only bind *Bax* directly to inhibit *Bax* activation but also bind the Apaf-1 apoptotic body to inhibit the apoptosis process.

In this study, to explore the possible molecular mechanism of *SNORD89* in endometrial cancer we performed in vitro and in vivo experiments.

## Results

### *SNORD89* expression is increased in endometrial carcinoma

**Fig. 1:**
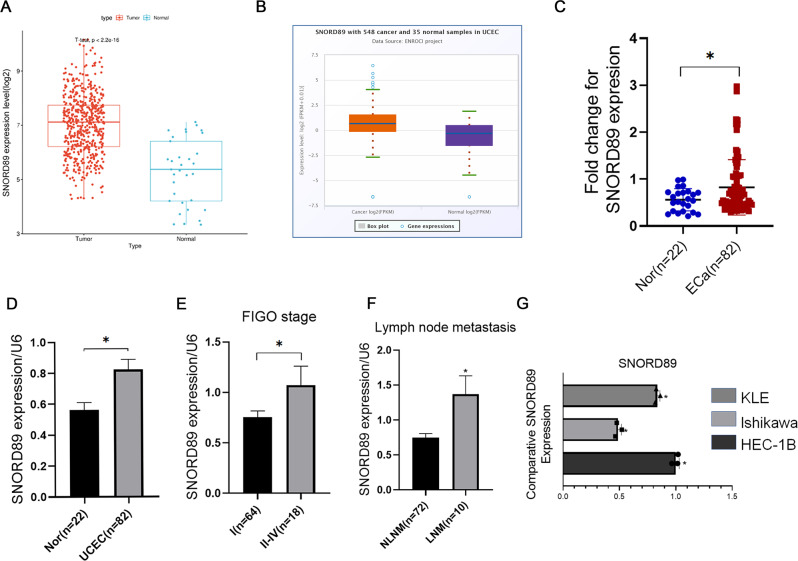
Gene expression levels. **A** Data from TCGA database; **B** Data from starBase; **C**, **D** *SNORD89* in tumoral (*n* = 82) and nontumoral (*n* = 22) endometrial tissue; **E** *SNORD89* in stage I (*n* = 64) and stage II-IV (*n* = 18); **F** *SNORD89* in tissues with lymph node metastasis, negative (*n* = 72), positive (*n* = 10). **G** *SNORD89* in EC cell lines (*n* = 3). Values are presented as the mean ± SD. *p ≤ 0.05; **p ≤ 0.01; ***p ≤ 0.001.

**Fig. 2:**
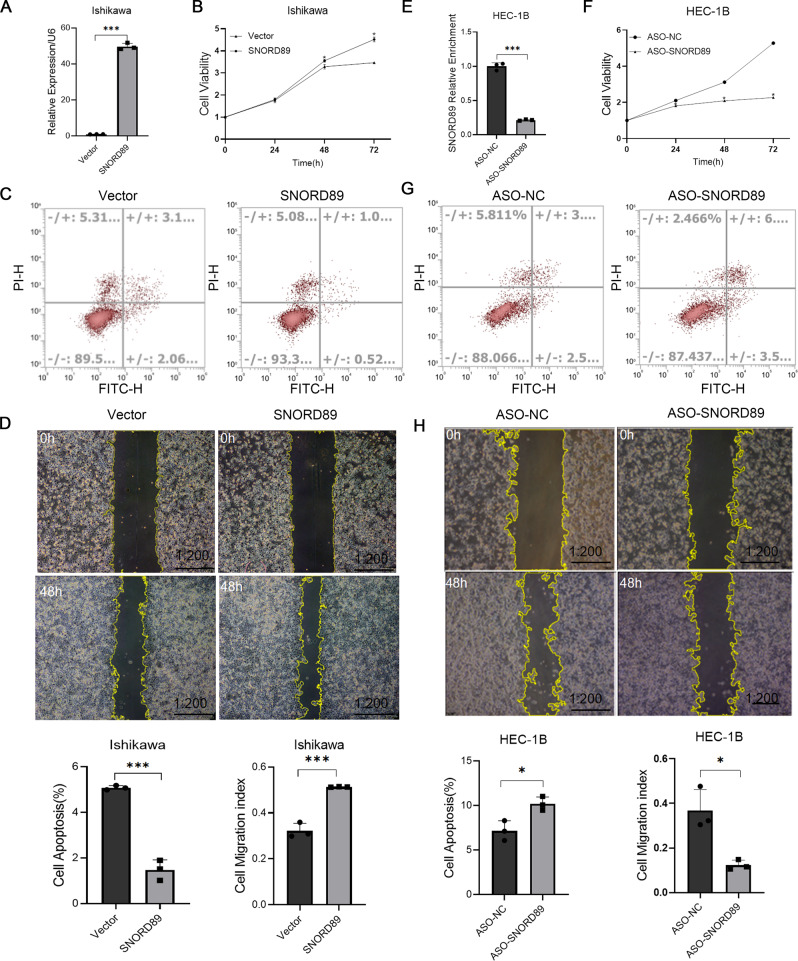
Effect of *SNORD89* on EC cells in vitro. **A** Transfection efficiency after overexpression of *SNORD89* (*n* = 3); **B** *SNORD89* overexpression induced cell viability (*n* = 3) and **D** cell migration (*n* = 3), **C** while reduced cell apoptosis (*n* = 3). **E** Transfection efficiency after downregulation of *SNORD89* (*n* = 3); *SNORD89* downregulation reduced **F** cell viability (*n* = 3), **H** cell migration (*n* = 3), while increased **G** cell apoptosis (*n* = 3). Values are presented as the mean ± SD. *p ≤ 0.05; **p ≤ 0.01; ***p ≤ 0.001.

In the TCGA database and starbase database analysis, the expression of *SNORD89* in endometrial cancer tissues was higher than that in normal endometrial tissues (Fig. 1A, B, *P < 0.05). qRT-PCR detected significantly increased expression of *SNORD89* in endometrial cancer tissues compared with its expression in normal endometrial tissues (Fig. 1C, D, *P < 0.05). In the analysis of the relationship between *SNORD89* expression level and clinicopathological features of endometrial cancer, the *SNORD89* expression level was higher in the International Federation of Gynecology and Obstetrics (FIGO) stage II-IV diseases than in FIGO stage I diseases (Fig. 1E, *P < 0.05). In addition, the *SNORD89* expression level was higher in endometrial carcinoma with lymph node metastasis than in endometrial carcinoma without lymph node metastasis (Fig. 1F, *P < 0.05). qRT-PCR analyses revealed that *SNORD89* expression was the lowest in Ishikawa cells, but the highest in HEC-1B cells (Fig. 1G, *P < 0.05). Therefore, Ishikawa cells were used for transfection of *SNORD89* overexpression plasmid, and HEC-1B cells were used for *SNORD89* ASO transfection. qRT-PCR was used to confirm the transfection efficiency of plasmid- or ASO-transfected cells (Fig. 2A, E, *P < 0.05).

### *SNORD89* overexpression promoted the proliferation and migration of endometrial cancer cells and inhibited apoptosis

Ishikawa cells were transfected with plasmids-overexpressing *SNORD89*. To evaluate the role of *SNORD89* in the proliferation, apoptosis, and migration of endometrial cancer cells, cell count, apoptosis, and wound-healing experiments were performed. The results revealed that *SNORD89* overexpression could promote cell proliferation (Fig. 2B, *P < 0.05), reduce apoptosis (Fig. 2C, *P < 0.05), and induce cell migration (Fig. 2D, *P < 0.05).

### The knockdown of *SNORD89* inhibited the proliferation and migration of endometrial cancer cells and promoted cell apoptosis

We transfected HEC-1B cells with ASO *SNORD89* and knocked down the expression level of *SNORD89* for the cell function experiment. As shown in Fig. 2F–H, *SNORD89* knockdown can inhibit cell proliferation and migration and promote cell apoptosis compared with the negative control group.

### In vivo effect of *SNORD89* overexpression

**Fig. 3:**
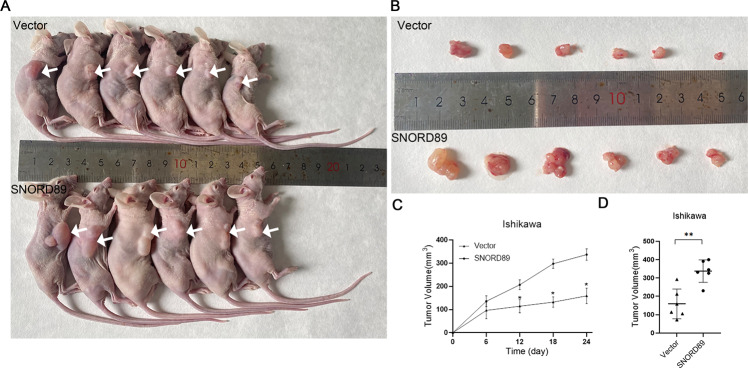
Effect of *SNORD89* overexpression on xenograft tumors in nude mice. **A**–**C** Tumorigenicity was greater in mice injected with the *SNORD89*-overexpressing cells than in vector; **D** The tumor volume was greater (*n* = 6). Values are presented as the mean ± SD. *p ≤ 0.05; **p ≤ 0.01; ***p ≤ 0.001.

Following the experiments detailed in the Materials and methods section, a subcutaneous tumor formation experiment was performed in nude mice to detect the in vivo effect of *SNORD89*. The results showed that compared with the control group, the tumor formation rate was faster, and the tumor volume was larger in mice injected with *SNORD89* Ishikawa-overexpressed cells (Fig. 3A–D, *P < 0.05).

### *SNORD89* downregulates Bim gene expression via Fbl

**Fig. 4:**
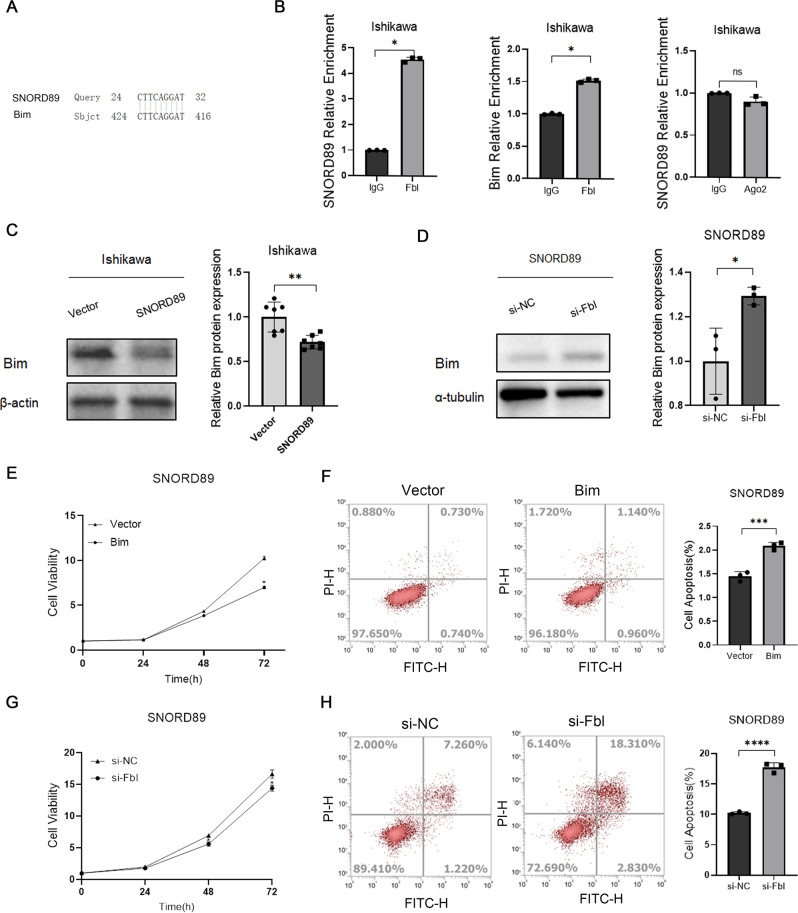
The relationship between *SNORD89*, *Bim*, and *Fbl*. **A** There are pairing points between *SNORD89* and *Bim*; **B** *Fbl* can bind *Bim* mRNA and *SNORD89*, but *SNORD89* cannot bind Ago2 (*n* = 3). **C** Overexpression of *SNORD89* in Ishikawa cells can reduce the protein level of *Bim* (*n* = 6); **D** Downregulation of *Fbl* can increase the overexpression of *Bim* protein in Ishikawa cells (*n* = 3); **E**–**H** downregulation of *Fbl* or overexpression of *Bim* can reverse the oncogenic effect of *SNORD89* (*n* = 3); Values are presented as the mean ± SD. *p ≤ 0.05; **p ≤ 0.01; ***p ≤ 0.001.

A possible binding site between the tumor suppressor gene *Bim* and *SNORD89* was found through the gene complementary pairing with *SNORD89* sequence base by bioinformatics prediction (NCBI database) (Fig. 4A). RIP experiments confirmed that *Bim* could combine with *SNORD89*, and the expression level of *Bim* protein decreased after *SNORD89* was overexpressed in Ishikawa cells (Fig. 4B, C, *P < 0.05). Meanwhile, we found that overexpression of Bim in *SNORD89*-overexpressed Ishikawa cells reverses the cell survival effect of *SNORD89*. (Fig. 4E, F, *P < 0.05). Therefore, it was hypothesized that *SNORD89* might play an oncogenic role in endometrial cancer by regulating *Bim*. *SNORD89* performs 2′-*O*-methylation modification mainly by binding to RNA-methylated proteins such as Fibrillarin (*Fbl*) or competitive endogenous RNA by binding to proteins such as Argonaute (AGO2). RIP experimental results showed that *SNORD89* could not bind AGO2 but could bind the *Fbl* protein (Fig. 4B, *P < 0.05). To further verify that *SNORD89* regulates Bim expression through Fbl, we knocked down Fbl in Ishikawa cells with overexpressed *SNORD89*, Fbl knockdown reversed the antiapoptotic effect of *SNORD89* (Fig. 4G, H, *P < 0.05), suggesting that in combination with Fbl *SNORD89* suppresses Bim gene expression.

### The relationship between *SNORD89* and Bim 2′-*O*-methylation was detected by the RTL-P experiment

**Fig. 5:**
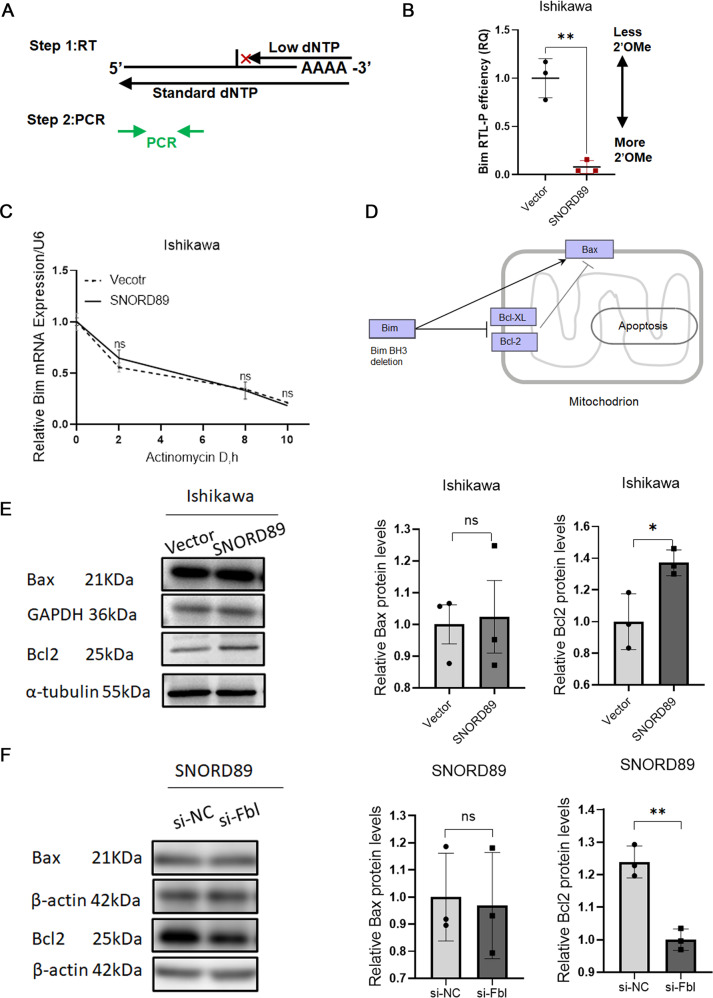
Changes of *Bim* mRNA and downstream pathways. **A** Illustration of the RTL-P method. The amount of qPCR product from the low dNTP reactions of two different samples can be compared to establish the relative “RTL-P efficiency (RQ)”. RQ relative quantity. See Methods for detail. **B** Under the low concentration of dNTPs, the Bim product was significantly reduced after overexpression of *SNORD89* and showed higher RTL-P efficiency. **C** *Bim* mRNA stability had no significant change compared with the vector group (*n* = 3). **D** *Bim*/Bcl-2/*Bax* was an important signaling pathway that mediated apoptosis. **E** Overexpression of *SNORD89* increased *Bcl2* protein level and *Bcl2* / *Bax* ratio (*n* = 3). **F** In Ishikawa cells overexpressing *SNORD89*, silencing *Fbl* reduced *Bcl2* protein and *Bcl2*/*Bax* ratio (*n* = 3). Values are presented as the mean ± SD. *p ≤ 0.05; **p ≤ 0.01; ***p ≤ 0.001.

In order to verify whether *SNORD89* combined with FBL regulates Bim expression by affecting the 2′-*O*-methylation modification level, we used the RTL-P experiment for detection, and the specific experimental method was obtained from existing literature. Figure 5A illustrates the principle of the RTL-P experiment, in which reverse transcriptase immediately stops cDNA synthesis before encountering 2′-*O*-methylated nucleotides at low concentrations of dNTPs. RTL-P experimental results showed that the Bim products of *SNORD89* overexpressed were significantly reduced at low concentration, suggesting higher 2′-*O*-methylation activity and more 2′-*O*-methylation sites (Fig. 5B, *P < 0.05).

### Relationship between *SNORD89* overexpression and *Bim* mRNA stability

We performed RNA stability tests in *SNORD89-*overexpressed Ishikawa cells, and the results confirmed that *SNORD89* overexpression did not affect the stability of *Bim* mRNA (Fig. 5C, *P < 0.05). The above results indicated that *SNORD89* combined with *Fbl* increased the level of 2′-*O*-methylation modification of *Bim* mRNA, affecting the complementary base pairing of *Bim* mRNA, thus reducing *Bim* protein.

### Changes in protein levels in endometrial cancer cell lines after *SNORD89* overexpression

The above results indicated that *SNORD89* regulated *Bim* 2′-*O*-methylation modification by binding *Fbl* to form a snoRNP complex, thus reducing the *Bim* protein level. Through the KEGG database (Fig. 5D), we found that *Bim*/*Bcl-2*/*Bax* was an important signaling pathway that mediated apoptosis. Western blotting showed that after *SNORD89* overexpression, the *Bcl-2* protein level and Bcl-2/*Bax* ratio increased (Fig. 5E, *P < 0.05). The results of the rescue experiment revealed that Bcl-2 protein level and *Bcl-2*/*Bax* ratio were decreased after *Fbl* silencing in Ishikawa cells with overexpressed *SNORD89* (Fig. 5F, *P < 0.05). The above results indicated that *SNORD89* was involved in the occurrence and development of endometrial cancer by combining *Fbl* to form a snoRNP complex and regulating the *Bim*/*Bcl-2*/*Bax* signaling pathway.

### *SNORD89* overexpression can decrease *Bim* protein level and increase Bcl-2 protein level in vivo

**Fig. 6:**
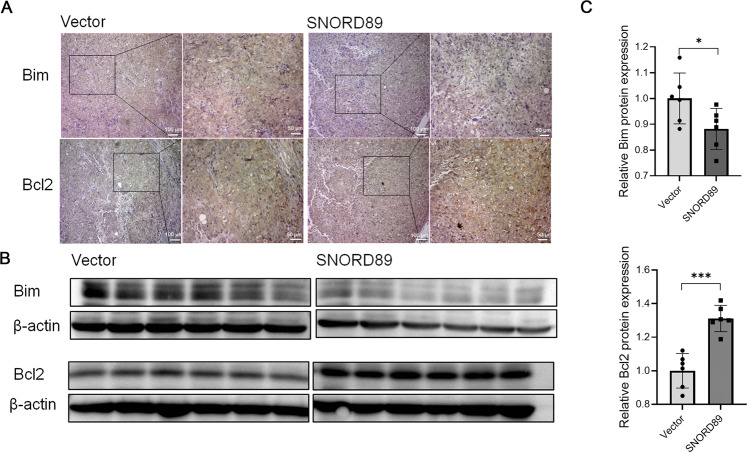
Effect of *SNORD89* overexpression on xenograft tumors in nude mice. **A**–**C** *SNORD89* overexpression can reduce the expression of *Bim* protein and increase the expression of *Bcl2* protein in vivo (*n* = 6). All results are representative of at least three independent experiments. Values are presented as the mean ± SD. *p ≤ 0.05; **p ≤ 0.01; ***p ≤ 0.001.

Immunohistochemical analysis of tumor tissues collected in vivo from subcutaneous tumorigenesis experiment in nude mice showed decreased expression of the *Bim* protein and increased expression of the *Bcl-2* protein in tumor tissues generated by Ishikawa cells with overexpressed *SNORD89* (Fig. 6A). By extracting tumor tissue proteins and conducting Western blotting experiments, the *Bcl-2* protein increased and *Bim* protein levels decreased in tumor tissues with overexpressed *SNORD89* (Fig. 6B, C, *P < 0.05). The above results further verified that *SNORD89* was involved in the occurrence and development of endometrial cancer by regulating the *Bim*/*Bcl-2*/*Bax* signaling pathway.

## Discussion

To search for diagnostic and therapeutic markers of endometrial cancer, we screened various snoRNAs from the TCGA database and found a significantly increased expression of *SNORD89* in endometrial cancer tissues compared with normal endometrial tissues. We further examined the expression of *SNORD89* in our clinical samples, the result of which was still the case. We speculated that it was related to the FIGO stage and lymph node metastasis and that *SNORD89* might be involved in the occurrence and progression of endometrial cancer and affect the prognosis.

To evaluate the function of *SNORD89* in endometrial cancer cells, we performed in vitro cell experiments and in vivo subcutaneous tumorigenesis experiments in nude mice. The transfection of *SNORD89*-overexpressing plasmids into Ishikawa cells with low *SNORD89* expression promoted cell proliferation, inhibited cell apoptosis, and induced cell migration. However, the cell experiment results were reversed after the expression level of *SNORD89* was knocked down in HEC-1B cells. In vivo subcutaneous tumorigenesis experiments in nude mice also confirmed that *SNORD89* overexpression can induce tumor growth in vivo. These results indicated that *SNORD89* had an oncogenic effect in endometrial cancer. However, the specific mechanism of action of *SNORD89* in endometrial cancer remained unclear.

We proved through the RIP experiment that *SNORD89* does not combine with AGO2, but with Fbl. Fbl silencing inhibited the proliferation of ovarian cancer cells with stable overexpression of *SNORD89*. Therefore, we believe that *SNORD89* may promote the development and progression of endometrial cancer by influencing the 2′-*O*-methylation modification of target genes.

BLAST search for downstream genes complementary to the *SNORD89* sequence revealed a binding point might exist between *SNORD89* and *Bim*. The RIP experiment confirmed that *Fbl* could bind with *Bim* and that *SNORD89* overexpression significantly reduced the expression of *Bim* protein. Therefore, we believe that *SNORD89* may be involved in the progression of endometrial cancer by regulating *Bim*. In order to verify that *SNORD89* plays its carcinogenic role through 2′-*O*-methylation modification of Bim mRNA, we designed corresponding primers and detected whether there was 2′-*O*-methylation modification at *SNORD89* and Bim pairing site by the RTL-P method. The results confirmed that Bim methylation modification was enhanced after overexpression of *SNORD89*.

2′-*O*-methylation modification refers to the addition of methyl at the 2′ positions of nucleic acid ribose so that the 2′-OH group is replaced by 2′-*O*-methyl [28]. This modification is mainly mediated by snoRNAs and has been confirmed to be involved in various cancers. For example, long ncRNA *ZFAS1* promotes snoRNA-mediated 2′-*O*-methylation through *Nop58* recruitment and is involved in the development and prognosis of colorectal cancer [29]. Methyltransferase *HENMT1* inhibits apoptosis of lung cancer cells by enhancing its stability by 2′-*O*-methylation of the 3′-terminal of *Mir-21-5p* [30]. Several studies have demonstrated that 2′-*O*-methylation modification was mainly located in rRNA [31]. Recently, some studies have shown that snoRNA could perform 2′-*O*-methylation modification on the mRNA of the target gene, and these modification sites could regulate mRNA and protein expression of the target gene [32].

2′-*O*-methylation modification can protect mRNA from hydrolysis, thus increasing the mRNA stability. However, this modification limits RNA chain conformation and suppleness, interferes with the ribosome monitoring base on the space and the homologous codon-anticodon spiral interaction, causes the excessiveness of homologous aminoacylation tRNAs in the initial selection and calibration to refuse, and inhibits the expression of target genes. Further, through RNA stability and Western blotting experiments, we confirmed that *SNORD89* do not alter the stability of Bim mRNA, but reduces the expression level of Bim protein. This suggests that *SNORD89* may promote the occurrence and development of endometrial cancer by binding *Fbl* to form a snoRNP complex 2′-*O*-methylation to modify *Bim* mRNA and reduce the expression level of *Bim* protein.

*Bim* is a member of the *Bcl-2* family. *Bcl-2* family proteins constitute key intracellular checkpoints and regulators of cell death and can inhibit or promote apoptosis. Irregularities in the members of the *Bcl-2* family are found in various cancers and diseases, including breast cancer [33] and prostate cancer [34]. Some studies have suggested that the intracellular *Bcl-2*/*Bax* protein ratio is the key to cell survival [35]. We found that the *Bcl-2*/*Bax* protein ratio increased significantly after *SNORD89* overexpression. Suggesting that *SNORD89* may affect downstream signaling pathways through *Bim*. Meanwhile, the rescue experiment showed that *Bim* protein increased after *Fbl* knockdown in the Ishikawa cell line stably transfected with overexpressed *SNORD89* plasmid. These results suggest that *Fbl* protein plays an important role in *SNORD89*’s regulation of *Bim*. Therefore, we believe that *SNORD89* may modify *Bim* mRNA through 2′-*O*-methylation, reduce the expression level of *Bim* protein, increase the ratio of *Bcl2* to *Bax*, and inhibit the activation of *Bax*, the release of mitochondrial *Cytc*, and subsequent apoptosis.

Finally, immunohistochemical analysis of tumor tissues collected from subcutaneous tumorigenesis experiment in nude mice confirmed that *SNORD89* overexpression in vivo could decrease the expression of *Bim* protein and increase the expression of *Bcl-2* protein in endometrial cancer cells. By extracting tumor tissue proteins and Western blotting experiments, the *Bcl-2* protein were increased, and the levels of *Bim* proteins were decreased in tumor tissues overexpressing *SNORD89*. *SNORD89* can be further verified by influencing *Bim* and its mediated *Bcl-2/Bax* signaling axis to participate in the occurrence and development of endometrial cancer.

## Conclusions

*SNORD89* can modify Bim through 2′-*O*-methylation and affect downstream signaling pathways to promote endometrial cancer occurrence and development. It enables snoRNA-mediated RNA 2′-*O*-methylation. The role of methylation modification in the prevention and treatment of endometrial cancer provides a new understanding and *SNORD89* may be a new diagnostic and therapeutic target for endometrial cancer.

## Materials and methods

### Bioinformatics analysis

The TCGA database (https://portal.gdc.cancer.gov/) and starbase database (http://starbase.sysu.edu.cn/) are used to analyze the expression level of *SNORD89* in normal endometrial tissues and endometrial cancer tissues. The NCBI database (https://www.ncbi.nlm.nih.gov/) was used to predict downstream target genes with complementary base pairs in *SNORD89*. The KEGG database (https://www.kegg.jp/) is used to query Bim mediated signal pathway.

### Endometrial cancer specimens

We collected 22 cases of normal endometrial tissues and 82 cases of endometrial cancer tissues from patients who underwent gynecological surgery in the Third Affiliated Hospital of Guangzhou Medical University. None of the patients received chemotherapy or radiotherapy before the operation. These tumor specimens were confirmed by two pathologists independently, then immediately frozen in liquid nitrogen and preserved at −80 °C until use. Informed consent was obtained from the subjects. The ethics committee of the Third Affiliated Hospital of Guangzhou Medical University approved the research (NO. 2020066), and all specimens were treated and anonymous according to ethical and legal standards.

### Cell culture and transformation

*SNORD89* expressions were screened from three human endometrial cancer cells (KLE, Ishikawa, and HEC-1B). Four human endometrial cancer cell lines were purchased from Jennio Biotech and cultured on Dulbecco’s Modified Eagle Medium (HEC-1B), Ham’s F-12 treatment medium (KLE), and RPMI 1640 medium (Ishikawa). All three media were supplemented with penicillin/streptomycin (100 U/mL) and 10% fetal bovine serum (FBS). All cell lines were recently STR identified and tested for mycoplasma contamination. Cells were cultured in an incubator with a constant temperature of 37 °C and 5% CO_2_. Lipofectamine 3000 (Invitrogen, Carlsbad, CA, USA) was used for plasmids, amido-bridged nucleic acid-flanked antisense oligonucleotides (ASOs), and siRNA transfection. An *SNORD89*-overexpressing plasmid was employed to upregulate *SNORD89* expression (ACTGAGGAATGATGACAAGAAAAGGCCGAATTGCAGTGTCTCCATCAGCAGTTTGCTCTCCATGGGCACACGATGACAAAATATCCTGAAGCGAACCACTAGTCTGACCTCAGT). A Bcl-2 interacting mediator of cell death (Bim)-overexpressing plasmid was adopted to upregulate Bim expression (ATGGCAAAGCAACCTTCTGATGTAAGTTCTGAGTGTGACCGAGAAGGTAGACAATTGCAGCCTGCGGAGAGGCCTCCCCAGCTCAGACCTGGGGCCCCTACCTCCCTACAGACAGAGCCACAAGGTAATCCTGAAGGCAATCACGGAGGTGAAGGGGACAGCTGCCCCCACGGCAGCCCTCAGGGCCCGCTGGCCCCACCTGCCAGCCCTGGCCCTTTTGCTACCAGATCCCCGCTTTTCATCTTTATGAGAAGATCCTCCCTGCTGTCTCGATCCTCCAGTGGGTATTTCTCTTTTGACACAGACAGGAGCCCAGCACCCATGAGTTGTGACAAATCAACACAAACCCCAAGTCCTCCTTGCCAGGCCTTCAACCACTATCTCAGTGCAATGGCTTCCATGAGGCAGGCTGAACCTGCAGATATGCGCCCAGAGATATGGATCGCCCAAGAGTTGCGGCGTATTGGAGACGAGTTTAACGCTTACTATGCAAGGAGGTTAGAGAAATAG), and an *SNORD89* ASO (CATGGGCACACGATGACAAA, Ruibo, Jiangsu, China) was applied to knockdown *SNORD89* expression. The siRNA sequence for Fbl (si-Fbl) knockdown was GGGCTAAGGTTCTCTACCT.

### Cell proliferation was detected by cell counting kit 8 (CCK8)

Cells were inoculated at 3000/well in 96-well plates, and transient transfection was performed after cell adherence. At specific time points (0, 24, 48, and 72 h), 10 μL of Cell Counting Kit 8 (CCK8, Yeasen, Shanghai, China) was added to each medium and cultured at 37 °C for 2 h. Then the absorbance was read at 450 nm. The experiment was repeated three times.

### Wound-healing assay

In this experiment, 6 × 10^5^ cells were seeded in 6-well plates. A vertical wound was marked in the Petri dish with the tip of a 200-μL pipette. Cells were cleaned three times with phosphate-buffered saline (PBS), and a 2-mL serum-free medium was added to the Petri dish. Wound healing was recorded at 0, 24, and 48 h. Image J software (National Institutes of Health, Bethesda, MD, USA) was used to determine the wound size and calculate the cell migration rate.

### Flow cytometry analysis of apoptosis

Apoptosis was detected by Annexin V-fluorescein isothiocyanate (FITC)/propidium iodide (PI) double staining according to the manufacturer’s protocol. Cells 3 × 10^5^/well were inoculated in six-well plates and cultured to a cell density of 60–70% for transient transfection. At 48 h after transfection, the cells were washed with cold PBS twice, digested, suspended with trypsin without ethylenediaminetetraacetic acid (1 × 10^6^ cells/mL), and cleansed with cold PBS three times. Cells were then cultured in 100 µL of binding buffer containing 5 µL of Annexin V-FITC and 5 µL of PI for 15 min in 4 °C darkness, with 400 µL of the binding buffer added to each tube. The cells were quantitatively analyzed by flow cytometry.

### Real-time quantitative reverse-transcription polymerase chain reaction (qRT-PCR)

An RNA Quick Purification kit was used to extract the total RNA from cells and tissues, and a Fast Reverse Transcription kit was used to generate endogenous complementary DNA. The cDNA was used as a template for qRT-PCR using the SYBR Prime X Ex-TAQ Patent II Suite (Takara Bio Inc., Shiga, Japan). Finally, the relative expression of the gene was calculated by comparing the period threshold (Ct) value of the target gene with the control gene (U6, encoding glyceraldehyde-3-phosphate dehydrogenase or 18sRNA gene) according to the 2−ΔCt method.

### RNA-binding protein immunoprecipitation (RIP) detection

TRIzol reagent (1 mL; Takamura, Shibuya, Japan) was used to extract the total RNA from cells or tissues. Then, 200 μL of chloroform was added to the extract, fully mixed, rested for 5 min, and centrifuged at 12,000 rpm for 20 min. The upper clarifying liquid was transferred to a new tube, and the RNA was precipitated with the same amounts of isopropyl alcohol. After centrifugation at 12,000 rpm for 20 min, the precipitate was retained and cleaned with 75% ethanol. Once fully dried at room temperature, sodium diethylpyrocarbonate (DEPC) water was used to dissolve the residue. The total RNA concentration was determined by spectrophotometry (Thermo Fisher Scientific, Shanghai, China). Then, using reverse transcriptase and random primers, 2 μg of RNA was reverse-transcribed into complementary DNA (cDNA) according to the manufacturer’s protocol (Yeasen). The cDNA was used as a template for qRT-PCR using the SYBR Prime X Ex-TAQ Patent II Suite (Takara Bio Inc., Shiga, Japan). Finally, the relative expression of the gene was calculated by comparing the period threshold (Ct) value of the target gene with the control gene (U6, encoding glyceraldehyde-3-phosphate dehydrogenase or 18sRNA gene) according to the 2−ΔCt method.

### RNA stability test

After transfection with overexpressed *SNORD89* plasmid, Ishikawa cells were treated with 5 μg/ml actinomycin D (ActD, CAS# A4262, Sigma). The total RNA was extracted from cells at 0, 2, 8, and 10 h after adding actinomycin D. qRT-PCR for analysis [36].

### Western blotting

Bim antibody (22037-1-AP, diluted 1:500; Proteintech, IL, USA), Bcl-2 (12789-1-AP, diluted at 1:1000; Proteintech), Bax (50599-2-LG, diluted at 1:5000; Proteintech), α-tubulin (11224-1-AP, diluted at 1:2000; Proteintech), and endometrial cancer cells were washed with PBS, homogenized in lysis buffer with RIPA containing protease inhibitors, and centrifuged at 12,000 rpm at 4 °C for 20 min. Protein concentration was determined by a BCA Protein Concentration Determination Kit (No. P0009; Beyotime Biotech Inc.). Protein lysates were separated on a 10% sodium dodecyl sulfate-polyacrylamide gel and transferred to Hybond membranes under appropriate conditions (Amersham, Munich, Germany). They were sealed at room temperature with Tris-buffered saline with Tween (TBST) containing 3% bovine serum albumin for 2 h. After sealing, primary antibodies Bim, Bcl-2, Bax, and imprinting were incubated overnight at 4 °C. On day 2, the membrane was incubated with a secondary antibody (1:8,000, Proteintech) at room temperature for 2 h, and α-tubulin was selected as the internal reference. After incubation, cell membranes were washed with TBST three times, using a hypersensitive ECL Chemiluminescence Kit (NcmECL UItra; Sliding Biotech, Shanghai, China) for protein development. The film was scanned using a full-function gel imaging system. Western blotting was quantified using Image J software.

### Subcutaneous tumorigenesis experiment in nude mice

The detection kit for subcutaneous tumor transmission was provided by Guangdong Medical Laboratory Animal Center (Guangdong, China) for *BALB/C* female nude mice, which were placed in a specific pathogen-free environment. The nude mice were randomly divided into groups according to the label. Ishikawa cells transfected with *SNORD89*/no-load plasmid (1 × 10^7^ in 150 μL of FBS-free medium) were subcutaneously injected into 5-week-old female mice to establish a tumor-forming nude mice model. After tumor formation, the tumor volume was measured weekly with vernier calipers to understand tumor growth. When the tumor reached a certain size, the mice were euthanized, the tumor nodules were resected, and their volume was measured. No blinding was done.

### Immunohistochemistry

The tumor tissue was collected by treating it with xylene and 100% ethanol and then decreasing the ethanol concentration. After antigen retrieval, endogenous peroxidase activity was blocked by incubation in 3% hydrogen peroxide for 40 min, then the slides were washed three times with PBS, and incubated in 5% bovine serum albumin for 40 min, stained with anti-BIM antibody (1:100, Proteintech, USA) and BCL-2 antibody (1:100, Proteintech, USA), followed by enzyme-conjugated secondary antibody (Dako) incubation, washed in PBS for 5 min, developed using DAB reagent. Hematoxylin is used for redyeing and images taken with light microscopes are gained.

### Reverse transcription at low dNTPs followed by PCR (RTL-P)

To detect the 2′-*O*-methylation (Nm) of *SNORD89*, RT-PCR was performed [32, 37]. The total reaction system was 20 μl. Containing 5 μg total RNA, a low (1 μM) or high (1 mM) concentration dNTPs (TaKaRa) and 1 μl specific RT primers (PF) were denatured at 65 °C for 5 min and then placed on ice. According to the instruction, 4 μl M-MLV RT 5×buffer (PROMEGA), 1 μl 200 u/μl M-MLV Reverse Transcriptase (PROMEGA), 1 μl 0.1 M DTT (PROMEGA), and 1 μl 40 u/μl RNase inhibitor (PROMEGA) were mixed at 50 °C for 1 h, and then heated at 70 °C for 15 min. cDNA is used as a template for QRT-PCR using the SYBR Prime X Ex-TAQ Patent II Suite (Takara). Finally, the relative expression of the gene was calculated by comparing the period threshold (Ct) of the target gene in the experimental group and the control group at low concentrations according to the 2−ΔCt method.

### Ethical statement

The animal study was reviewed and approved by Guangdong Provincial Medical Experimental Animal Center (Approval no. S2021-101), with a license to use experimental animals [SYXK(Guangdong)2018–0002]. Written informed consent was obtained from the owners for the participation of their animals in this study.

### Data analysis

GraphPad Prism (version 8.02(263)) was used for graphics and data analysis. Relevant data were expressed as mean ± standard mean error. Statistical analysis was performed using two-sample *t*-test and *P* < 0.05 was considered to indicate a significant difference.

## Supplementary information

Original full-length western blots

Supplementary materials

## Data Availability

The data generated or analyzed during this study are included in this published article and its supplementary information files, Supplementary information is available at Cell Death Discovery’s website.
