# Supplementary figures and images for: Box C/D snoRNA SNORD89 influences the occurrence and development of endometrial cancer through 2’-O-methylation modification of Bim
Source: Cell Death Discov. 2022 Jul 5;8:309. doi: 10.1038/s41420-022-01102-5 (PMC9256700; doi:10.1038/s41420-022-01102-5)

Figure 4C

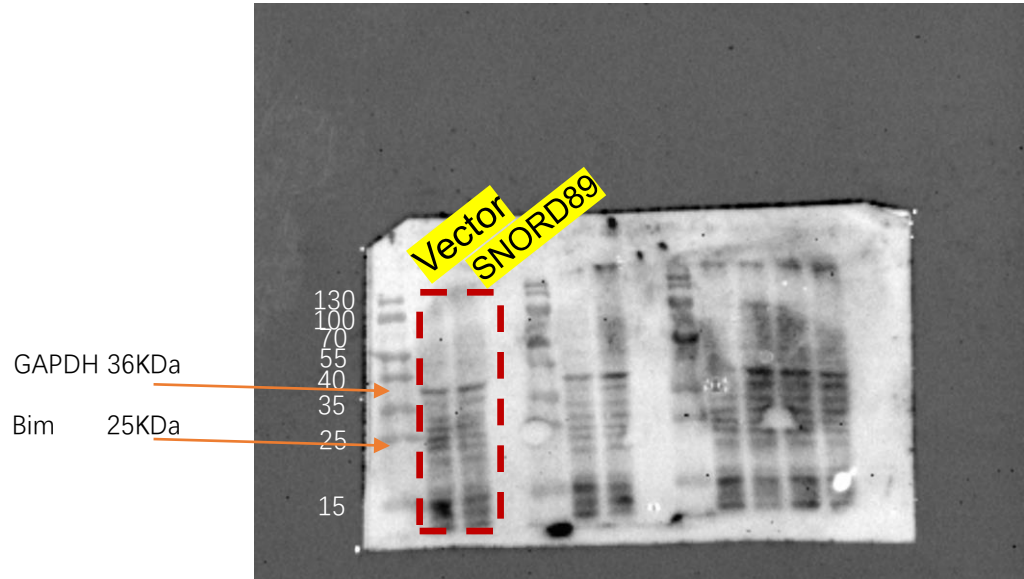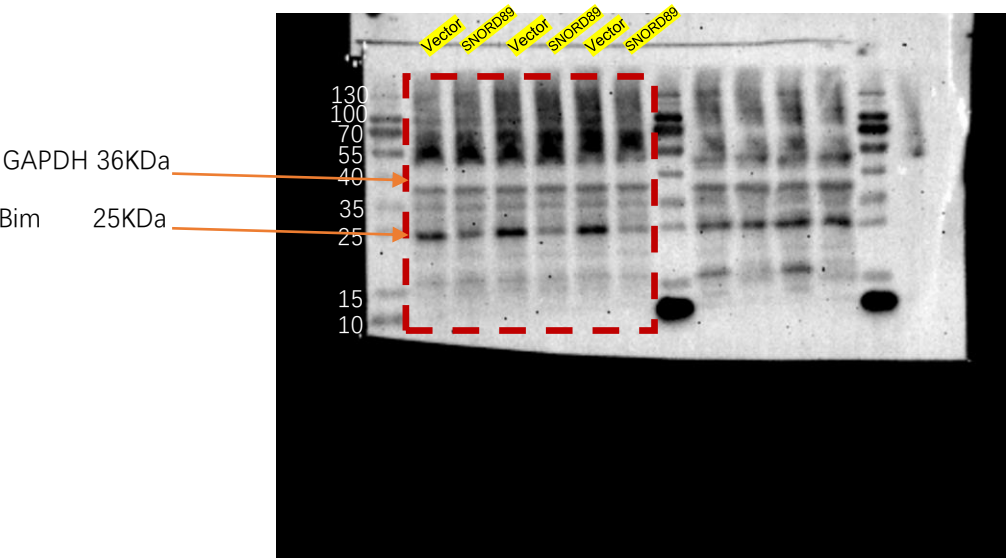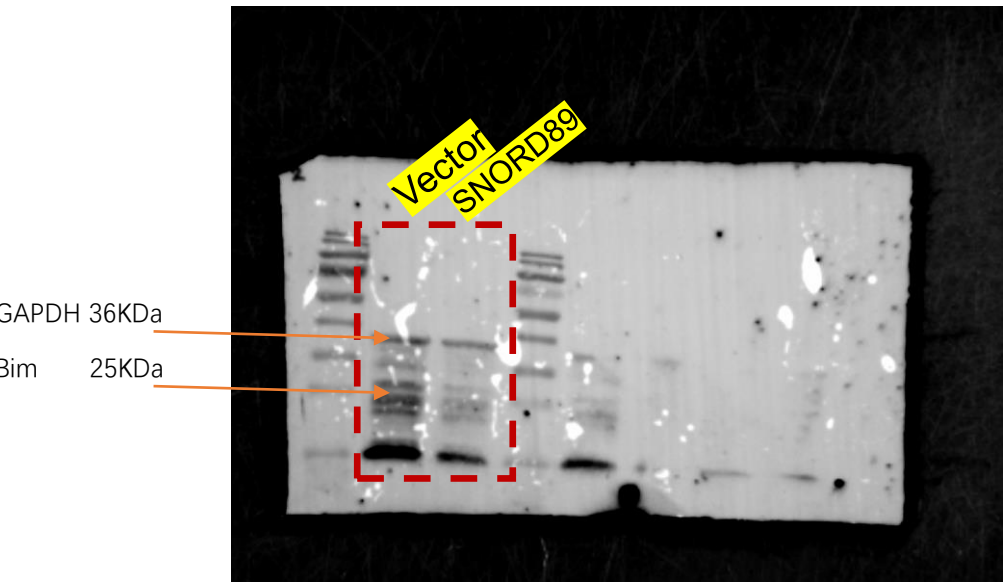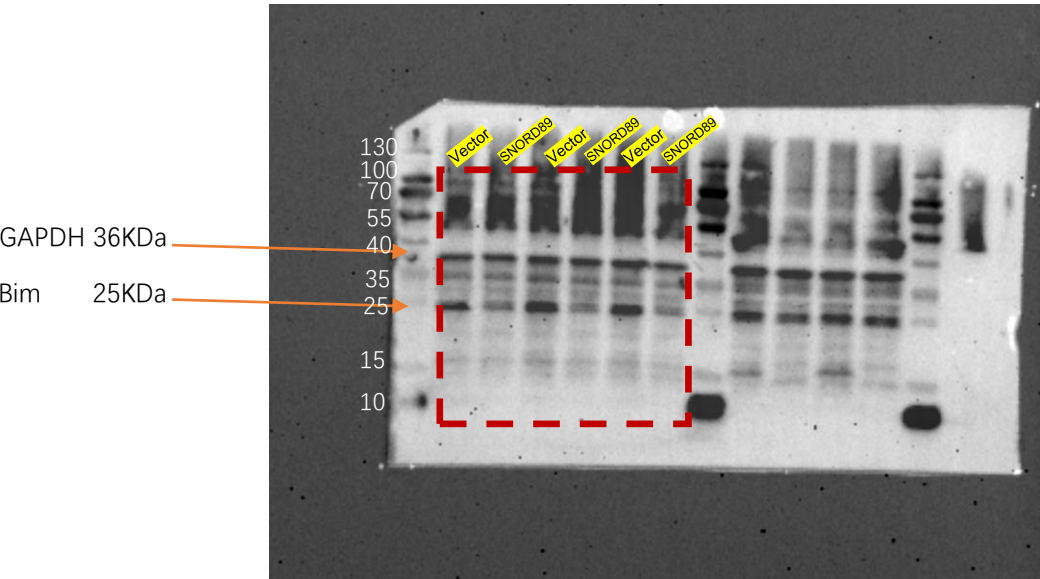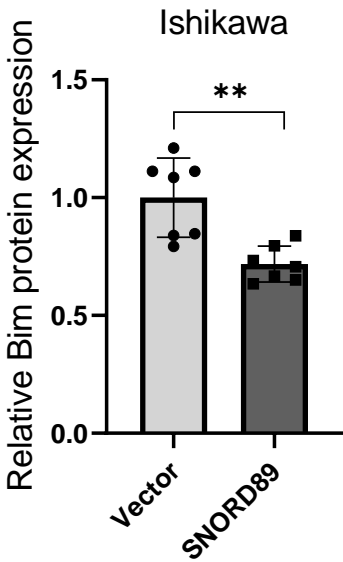

Figure 5E

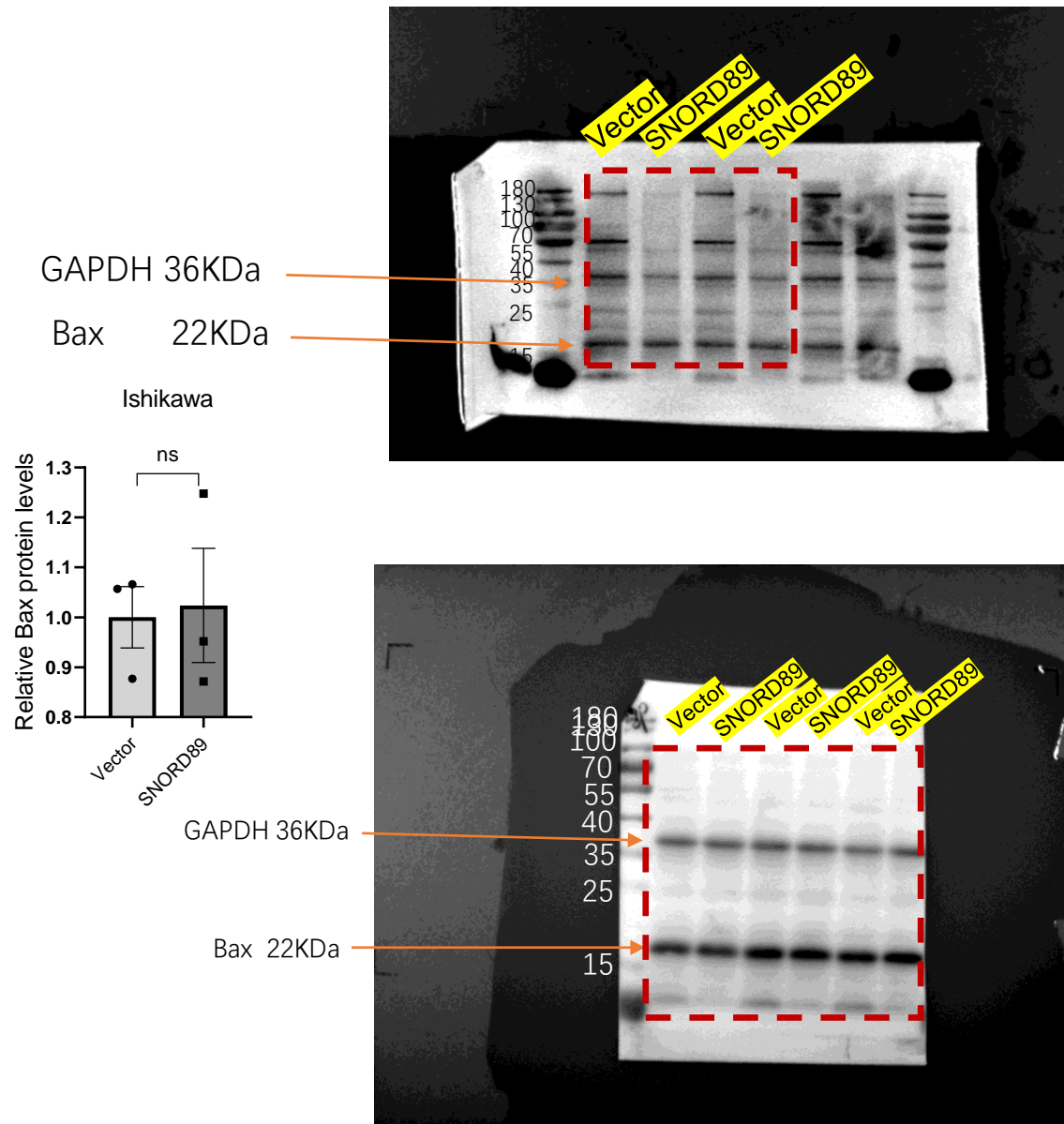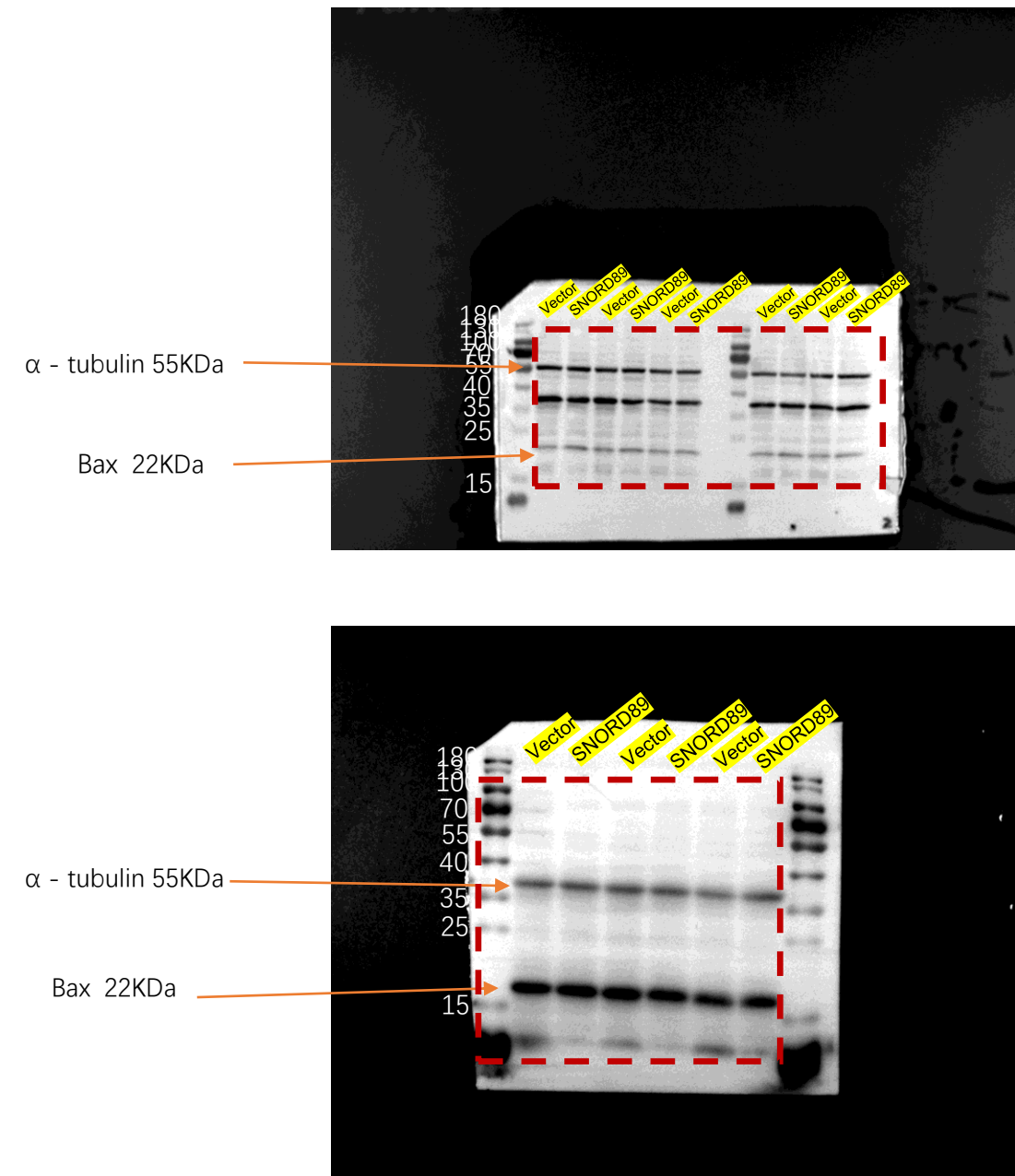

Figure 5E

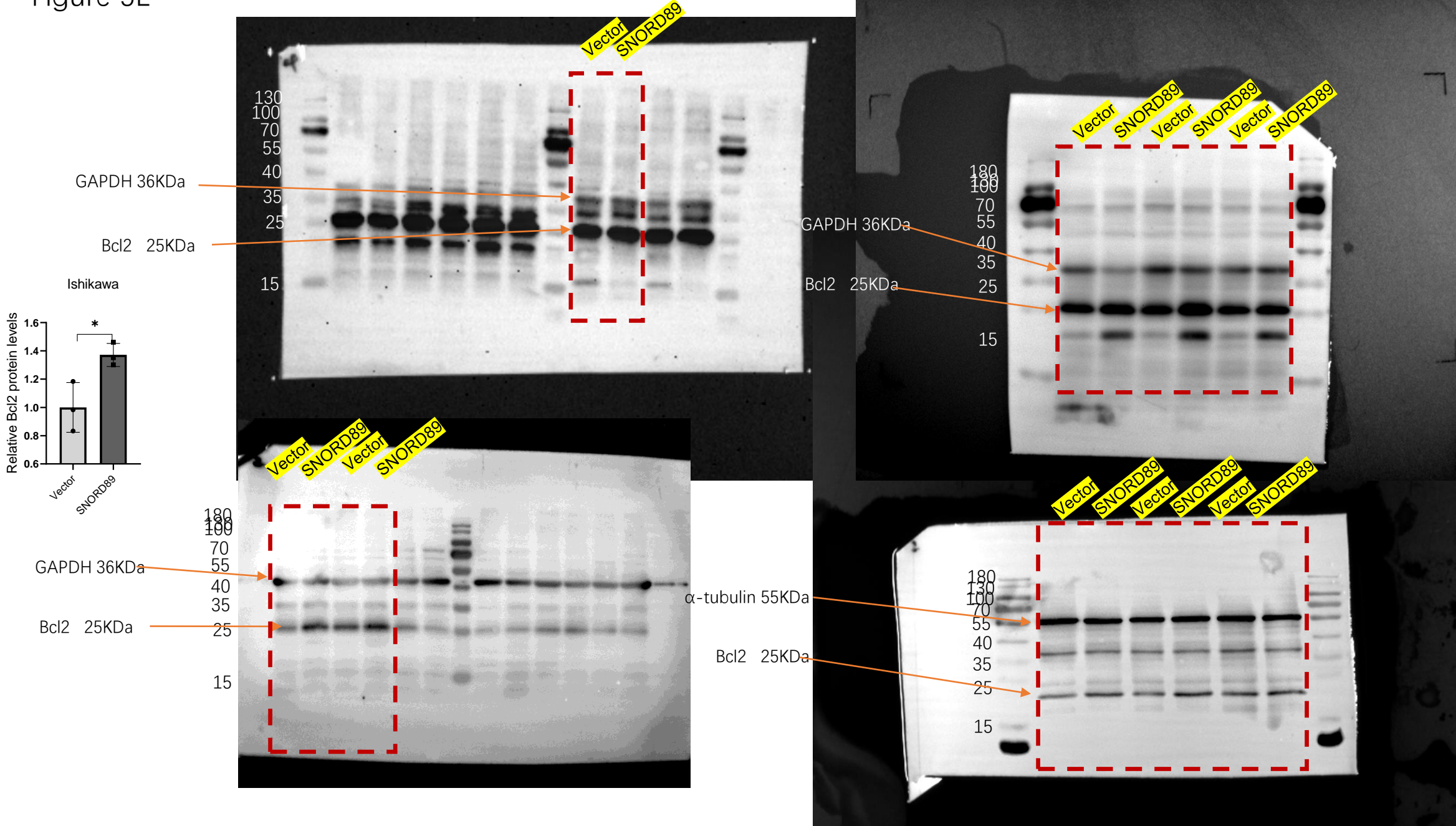

Figure 4D

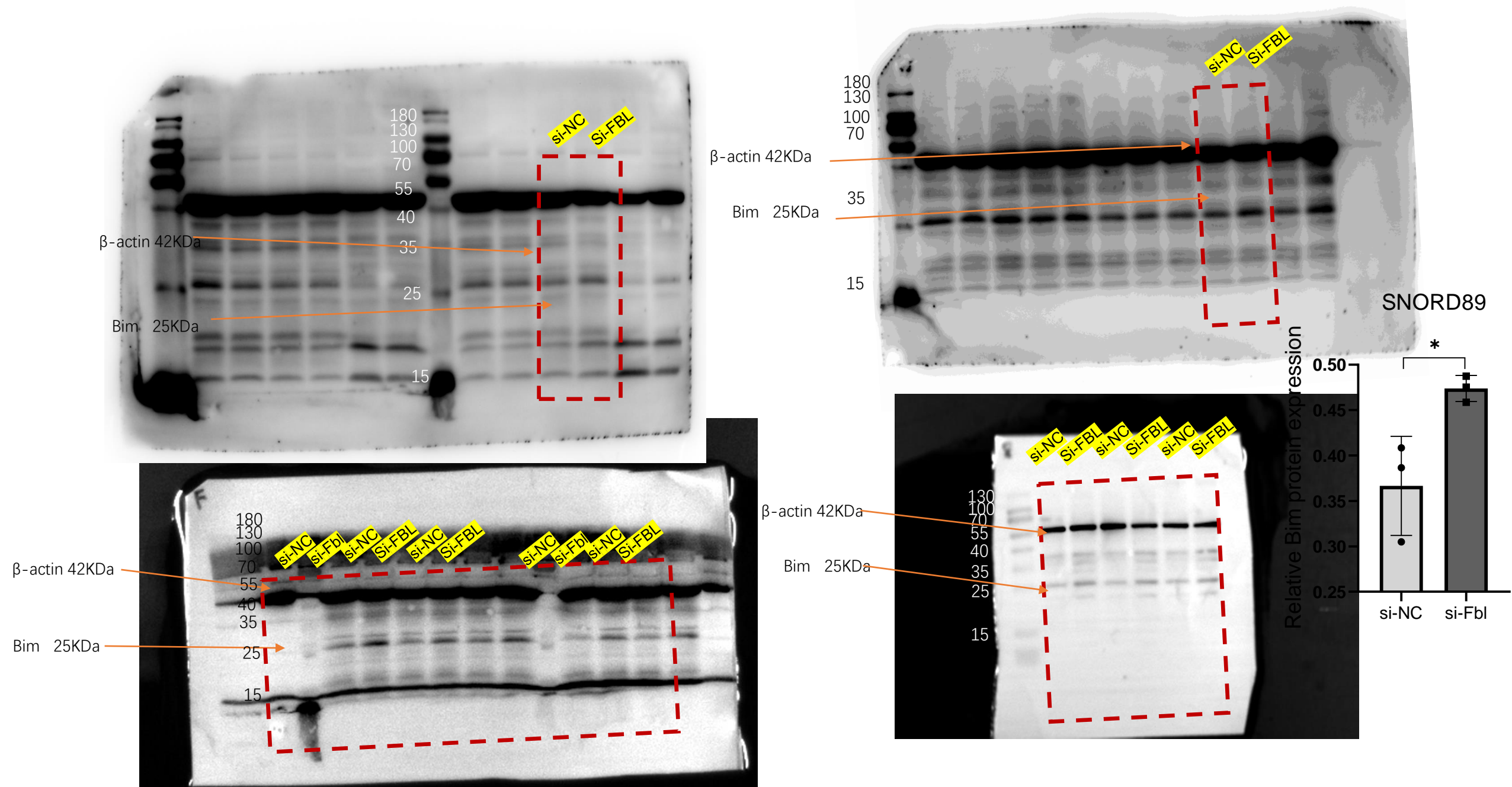

Figure 5F

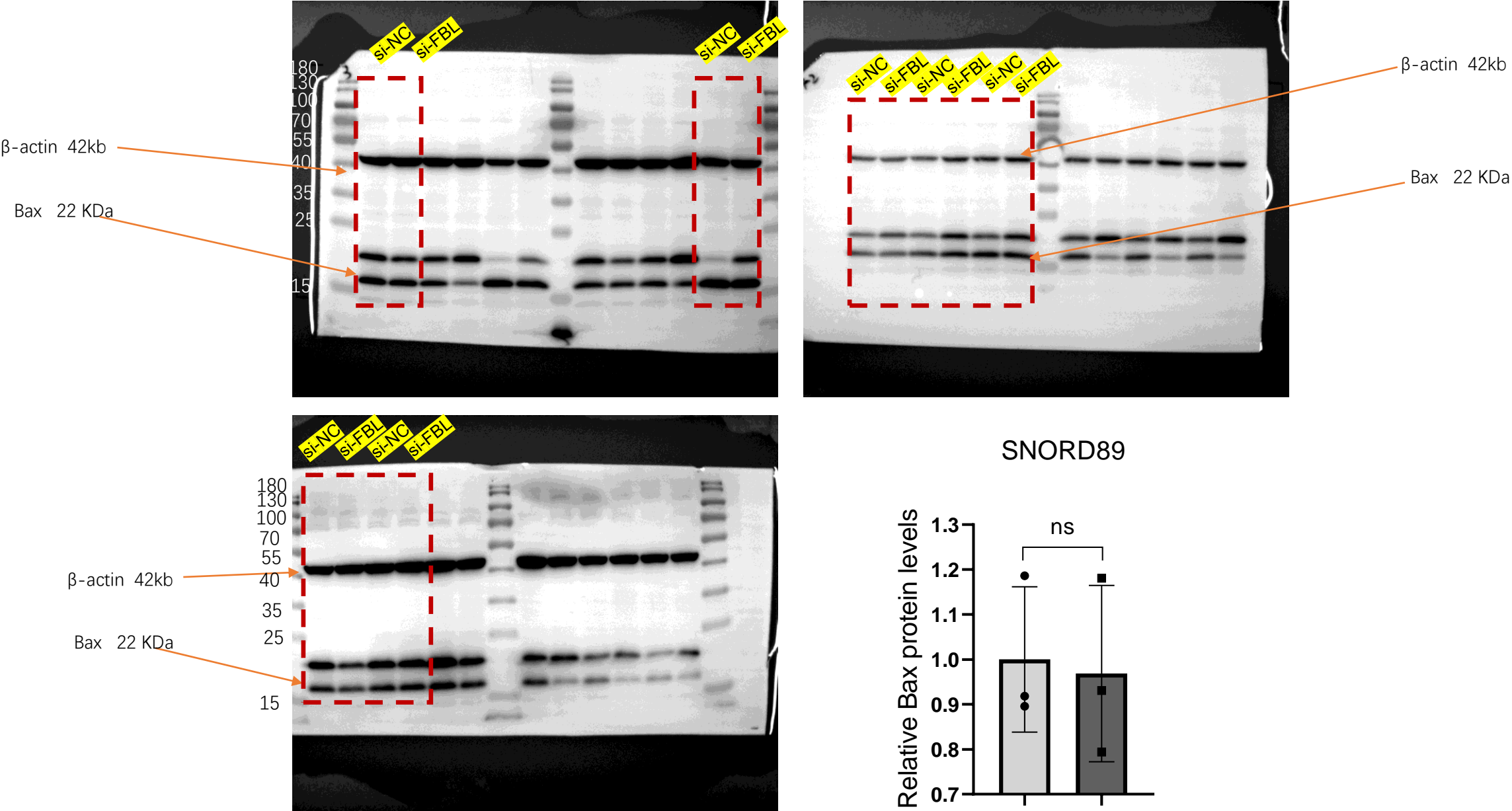

Figure 5F

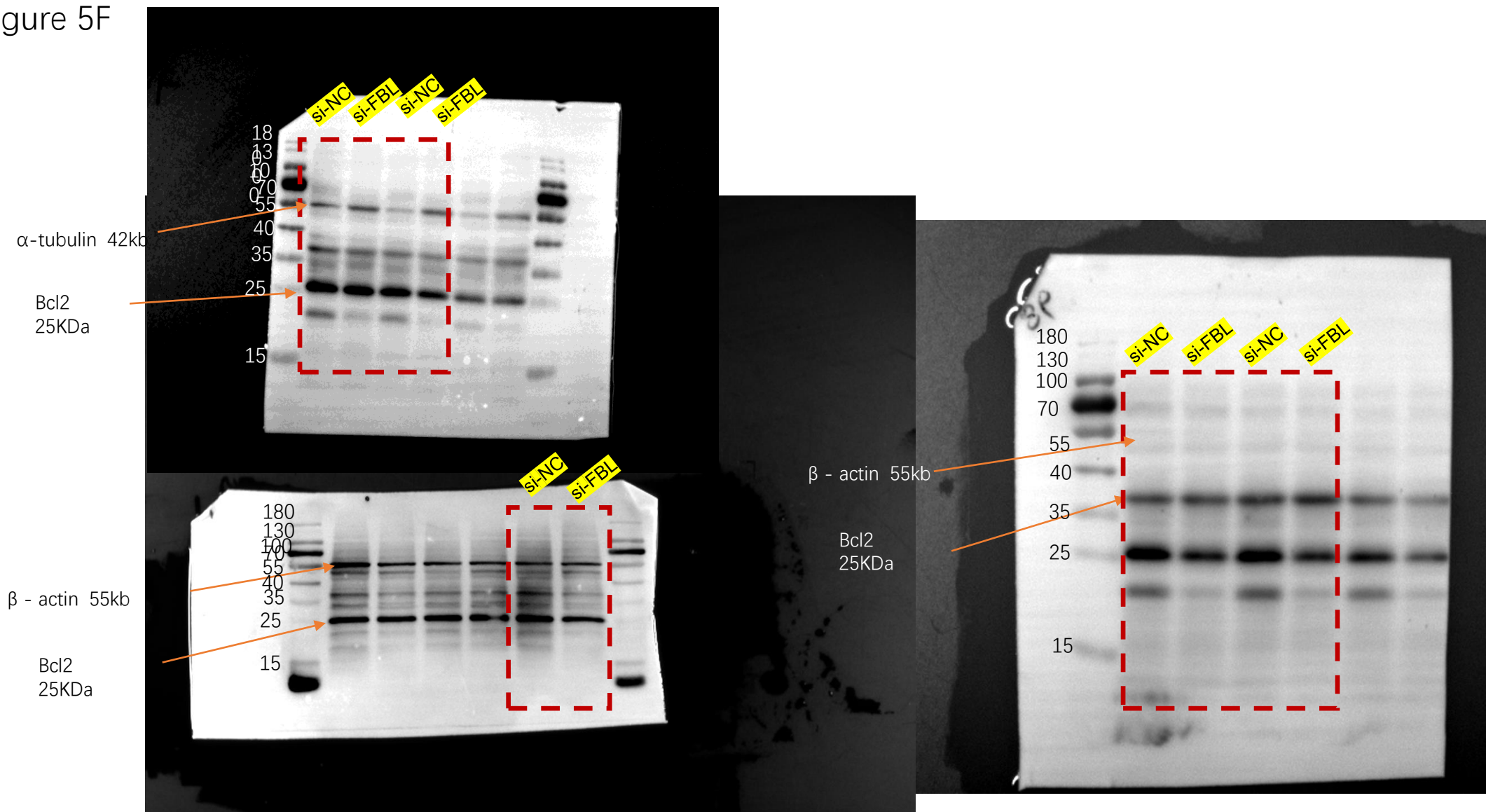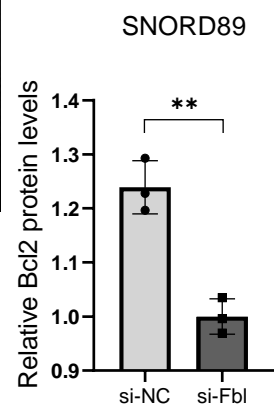

Figure 6B

Mice bim

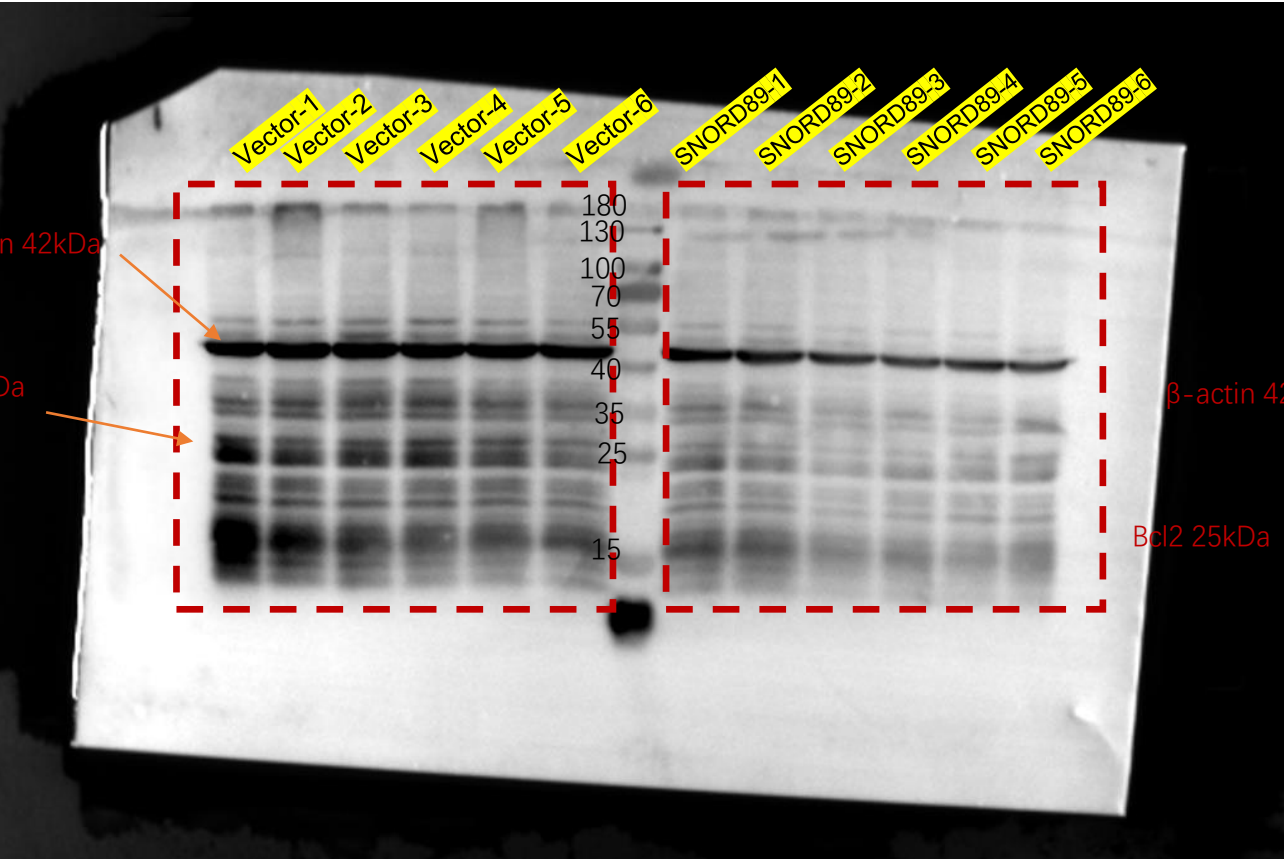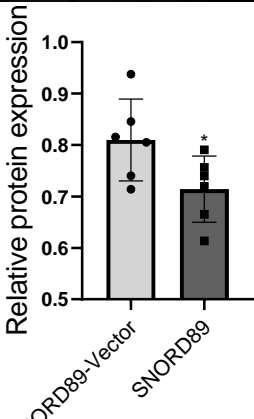

Mice bcl2

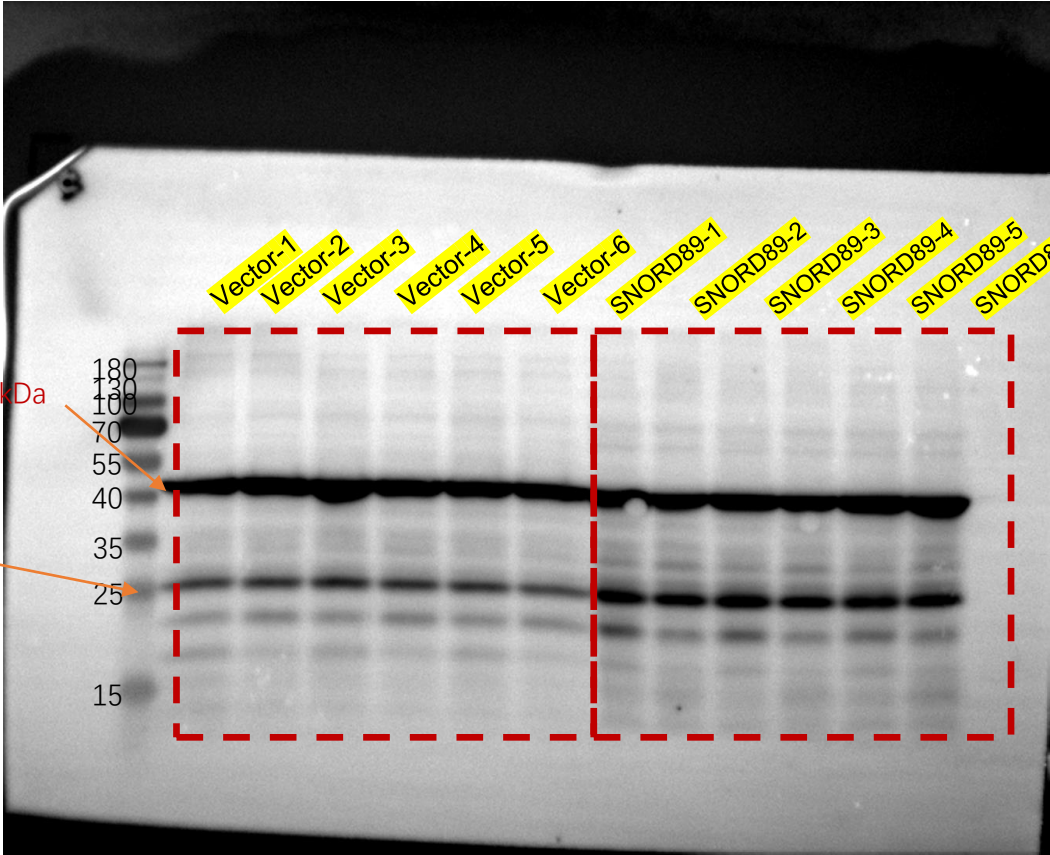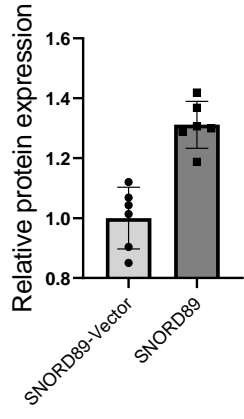

Supplement: Supplementary file 1 — Original full-length western blots [file 41420_2022_1102_MOESM1_ESM.pdf]
