# Supplementary material for: Box C/D snoRNA SNORD89 influences the occurrence and development of endometrial cancer through 2’-O-methylation modification of Bim
Source: Cell Death Discov. 2022 Jul 5;8:309. doi: 10.1038/s41420-022-01102-5 (PMC9256700; doi:10.1038/s41420-022-01102-5)
Supplement: Supplementary file 2 — Supplementary materials [file 41420_2022_1102_MOESM2_ESM.docx]

**Supplementary Table 1:** SNORD89 expression in Endometrial carcinoma

| **Groups** | **N** | **SNORD89 expression / U6** | | ***P* value** |
| --- | --- | --- | --- | --- |
|  |  |  |  |  |
| Normal Endometrial | 22 | | 0.823734523 ± 0.590039282 | ***0.0310949*** |
| Endometrial carcinoma | 82 | 0.542040036 ± 0.236839502 | |  |

Bold and Italics means P < 0.05.

**Supplementary Table 2:** Correlation of SNORD89 expression with different clinicopathological features of Endometrial carcinoma

| **Clinicopathological features** | **N** | **SNORD89 expression / U6** | ***P* value** |
| --- | --- | --- | --- |
|  |  |  |  |
| **The pathology types** |  |  | 0.600 |
| Adenocarcinoma | 75 | 0.417812548 ± 0.563591768 |  |
| The other pathology types | 7 | 0.553331829 ± 0.875385619 |  |
| **Age** |  |  | 0.943 |
| ＜ 45 | 9 | 0.810392765 ±0.601168921 |  |
| ≥ 45 | 73 | 0.825379398 ±0.592860563 |  |
| **FIGO stages** |  |  | ***0.042*** |
| I | 64 | 0.753681598 ± 0.503884784 |  |
| II-IV | 18 | 1.07281159 ± 0.796070467 |  |
| **Pathology classification** |  |  | ***0.001*** |
| Well | 72 | 0.747835421 ± 0.511288041 |  |
| Mod + Poor | 10 | 1.370208056 ± 0.832549978 |  |
| Bold and Italics means P < 0.05. | | | |

**Supplemental Table 3:** SNORD89 expression plasmid construction

ACTGAGGAATGATGACAAGAAAAGGCCGAATTGCAGTGTCTCCATCAGCAGTTTGCTCTCCATGGGCACACGATGACAAAATATCCTGAAGCGAACCACTAGTCTGACCTCAGT

**Supplemental Table 4**: Bim expression plasmid construction

ATGGCAAAGCAACCTTCTGATGTAAGTTCTGAGTGTGACCGAGAAGGTAGACAATTGCAGCCTGCGGAGAGGCCTCCCCAGCTCAGACCTGGGGCCCCTACCTCCCTACAGACAGAGCCACAAGGTAATCCTGAAGGCAATCACGGAGGTGAAGGGGACAGCTGCCCCCACGGCAGCCCTCAGGGCCCGCTGGCCCCACCTGCCAGCCCTGGCCCTTTTGCTACCAGATCCCCGCTTTTCATCTTTATGAGAAGATCCTCCCTGCTGTCTCGATCCTCCAGTGGGTATTTCTCTTTTGACACAGACAGGAGCCCAGCACCCATGAGTTGTGACAAATCAACACAAACCCCAAGTCCTCCTTGCCAGGCCTTCAACCACTATCTCAGTGCAATGGCTTCCATGAGGCAGGCTGAACCTGCAGATATGCGCCCAGAGATATGGATCGCCCAAGAGTTGCGGCGTATTGGAGACGAGTTTAACGCTTACTATGCAAGGAGGTTAGAGAAATAG

**Supplemental Table 5**: The ASO sequence for SNORD89 and the siRNA sequence for Fbl

| Names | Sequences |
| --- | --- |
| ASO-SNORD89 | CATGGGCACACGATGACAAA |
| si-Fbl | GGGCTAAGGTTCTCTACCT |

**Supplemental Table 6**: The detail of primer sequence

| Names | Sequences |
| --- | --- |
| SNORD89 primer | F: 5’ GTCTCCATCAGCAGTTTG 3’ |
|  | R: 5’ TGGTTCGCTTCAGGAT 3’ |
| Bim primer  RTL-P：Bim primer | F: 5’ GTATTCGGTTCGCTGCGTTC 3’ |
|  | R:5’ ACCTCCGTGATTGCCTTCAG3’  F:5’CCCTTGTGGACAACTGTGAGTA3’  R:5’CAATGCTGTTTAGAGTAGACCC3’ |
